# Supplementary figures and images for: Flavin affinity for the reductase HpaC differentially sensitizes Neisseria gonorrhoeae during Type IV pilus-dependent killing
Source: PLoS Pathog. 2025 Oct 27;21(10):e1013607. doi: 10.1371/journal.ppat.1013607 (PMC12558477; doi:10.1371/journal.ppat.1013607)

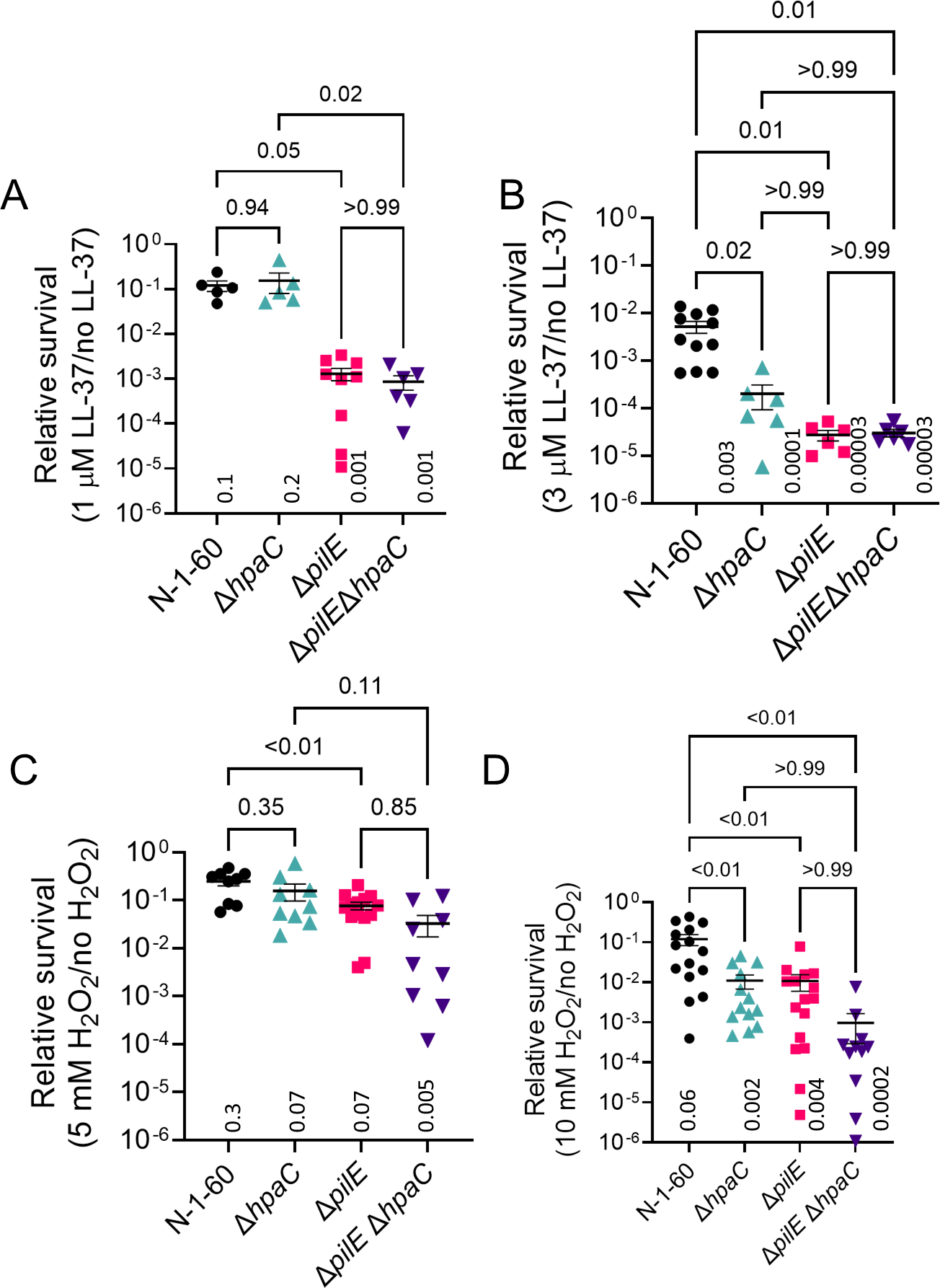

Supplement: S1 Fig — Relative survival of FA1090 (N-1–60), ΔhpaC::ERB (N-8–59), ΔpilE (N-1–69), and ΔpilEΔhpaC (N-8–61) to LL-37 (A and B) and hydrogen peroxide (C and D). A one-way ANOVA was used to determine statistical significance. The means and SEMs are plotted for biological replicates. (TIF) [file ppat.1013607.s001.tif]

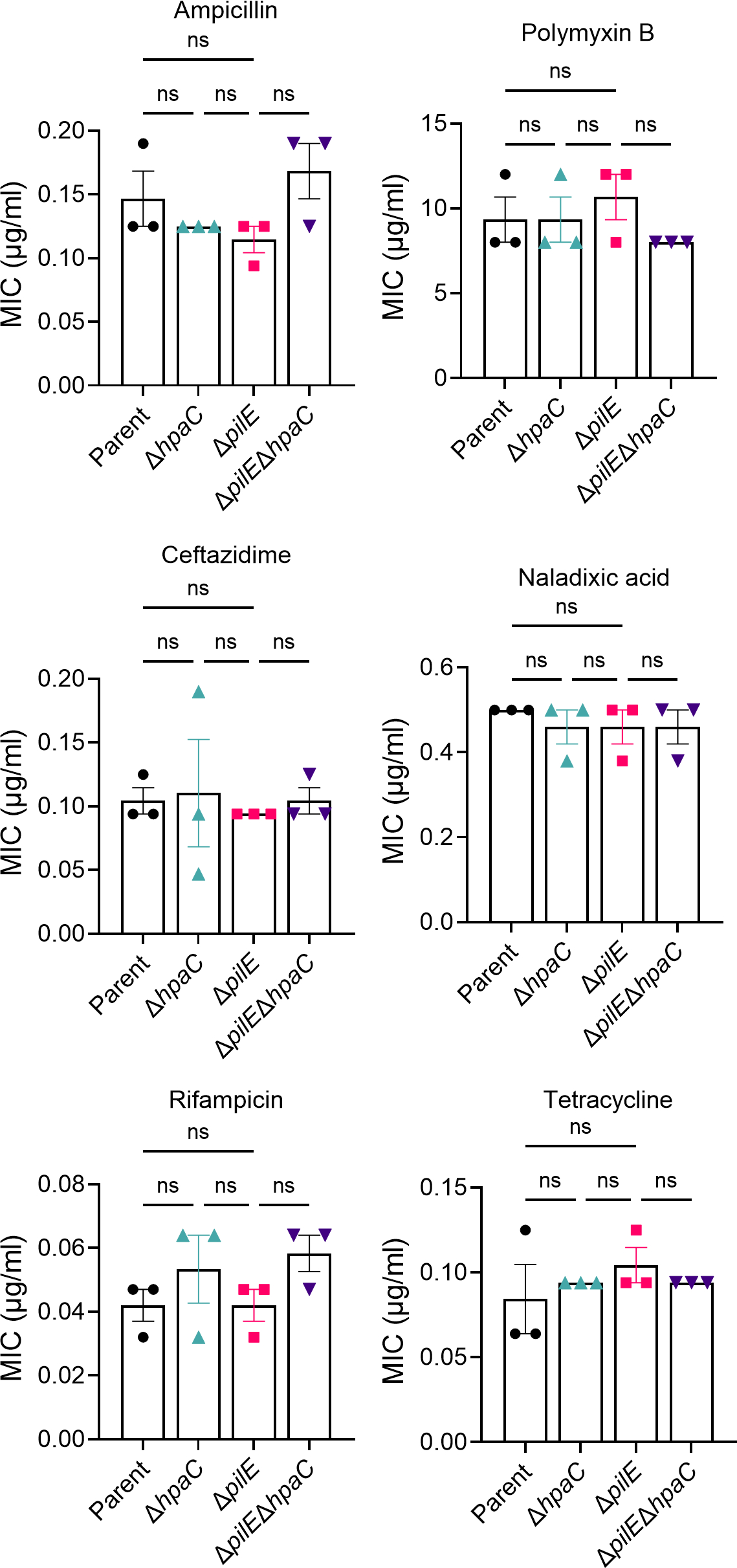

Supplement: S2 Fig — The minimum inhibitory concentrations for six antimicrobials were determined using E-test strips (µg/ml). Three biological replicates of the parental strain (N-1–60) and isogenic mutants ΔpilE (N-1–69), ΔhpaC (N-8–59), and a double mutant ΔpilEΔhpaC (N-8–61) were tested. Statistical significance was determined by a one-way ANOVA followed by a Sidak’s multiple comparisons test. (TIF) [file ppat.1013607.s002.tif]

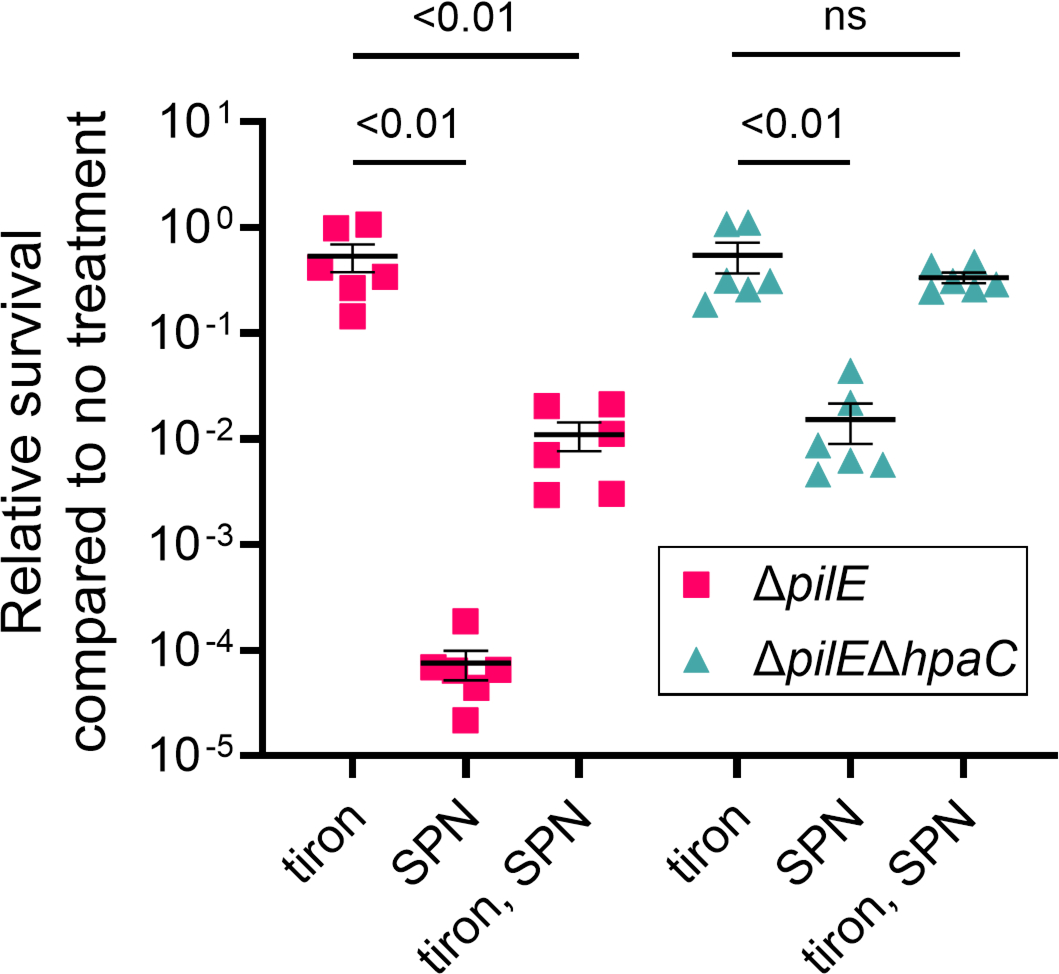

Supplement: S3 Fig — Average relative survival is shown with standard error of the mean for six biological replicates. A 2way ANOVA followed by a Dunnett’s multiple comparisons test was used to determine statistical significance. (TIF) [file ppat.1013607.s003.tif]

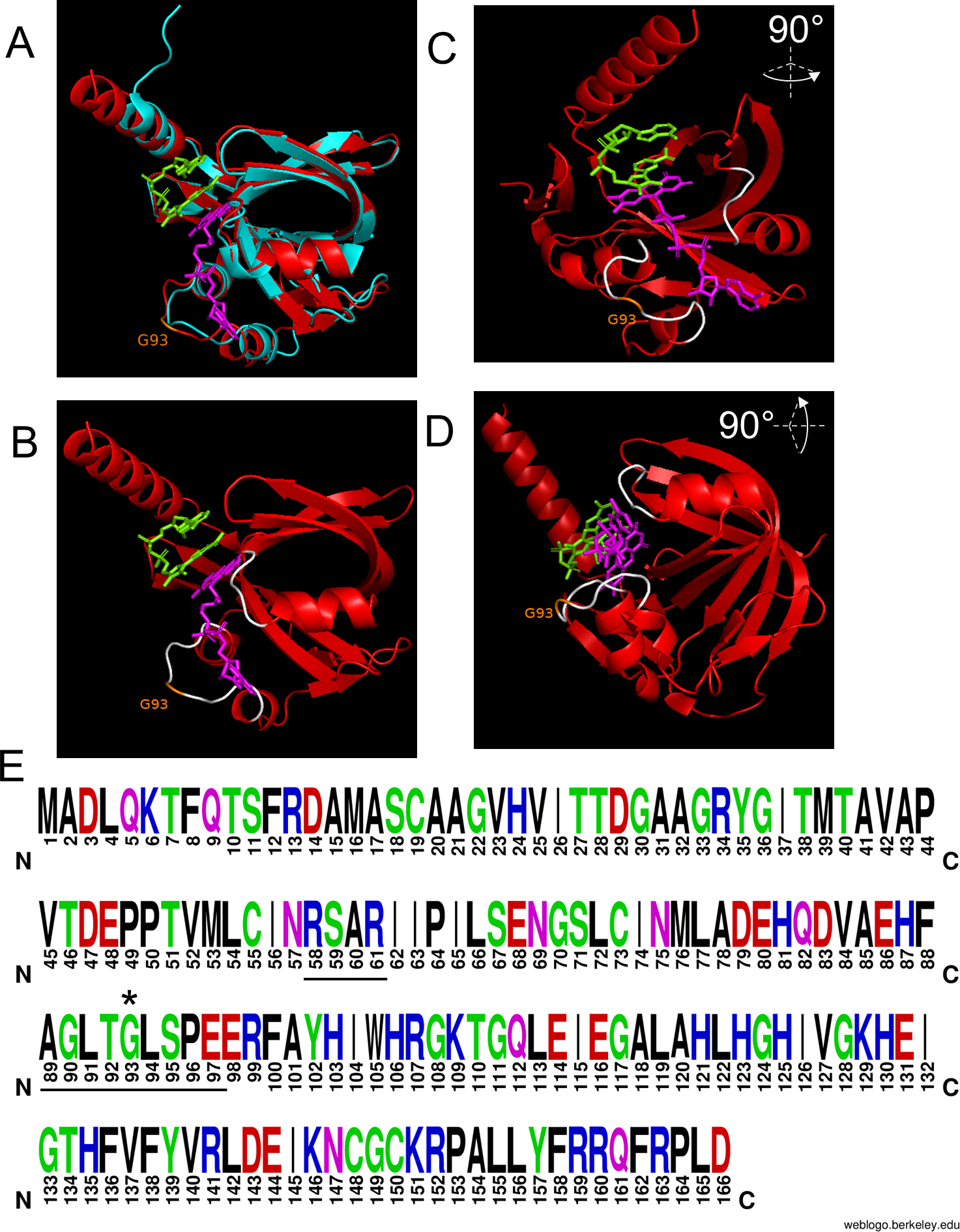

Supplement: S4 Fig — A) Overlay of B. cepacia TftC (PDB 3K88 in cyan) and FA1090 HpaC (red). Glycine-93 is highlighted in orange. B-D) FA1090 HpaC modeled with FAD (purple) and NADH (green) and the two conserved FAD binding loops (white). HpaC from B is rotated 90° to the right (C) or 90° up (D) to show different perspectives of the FAD binding pocket. E) N. gonorrhoeae HpaC consensus logo sequence. A consensus sequence of 999 NEIS0375 HpaC homologs with a peptide sequence of 166 residues from PubMLST using weblogo.berkley.edu. Underlined are the two conserved FAD binding loops. Marked with an asterisk is amino acid residue 93. (TIF) [file ppat.1013607.s004.tif]
