# Supplementary material for: Flavin affinity for the reductase HpaC differentially sensitizes Neisseria gonorrhoeae during Type IV pilus-dependent killing
Source: PLoS Pathog. 2025 Oct 27;21(10):e1013607. doi: 10.1371/journal.ppat.1013607 (PMC12558477; doi:10.1371/journal.ppat.1013607)
Supplement: S1 Table — After in vitro evolution, reads from whole genome sequencing were aligned to an unevolved reference strain N-1–60 [19] and variants unique to the evolved ΔpilE mutant were called. (PDF) [file ppat.1013607.s005.pdf]

S1 Table. Variant calling after streptonigrin in vitro evolution in Δ*pilE*

INTRAGENIC CHANGES

**Locus**	**Gene**	**Mutation**	**Product**
NGO0059	*hpaC*	missense variant: G277T; Gly93Cys	4-hydroxyphenylacetate 3-monooxygenase, reductase component HpaC
NGO0650	*recN*	synonymous variant: G597T; Ala199Ala	ATP-dependent RNA helicase RecN
NGO11100	*opaB*	phase variant: (CTTCT)8→9	OpaB opacity protein
NGO09965	*opaE*	phase variant: (CTTCT)13→14	OpaE opacity protein
NGO1765	*pglA*	phase variant: (G)9→10	pilin glycosyl transferase family 1
NGO2158	*lgtD*	phase variant: (G)10→14	glycosyltransferase LgtD

INTERGENIC CHANGES

**Between loci**	**Mutation**	**Description**
NGO2093 - NGO2094	phase variant: (C)9→12	-108 bp FetA/-396 bp molecular chaperone GroES
NGO1810 - NGO1811	phase variant: (C)10→9	+767 bp hypothetical protein/-103 bp tRNA pseudouridine synthase TruA
