# Supplementary material for: Flavin affinity for the reductase HpaC differentially sensitizes Neisseria gonorrhoeae during Type IV pilus-dependent killing
Source: PLoS Pathog. 2025 Oct 27;21(10):e1013607. doi: 10.1371/journal.ppat.1013607 (PMC12558477; doi:10.1371/journal.ppat.1013607)
Supplement: Attachment — Submitted filename: Response_to_Reviewers.pdf [file ppat.1013607.s007.pdf]

Response to reviewers' comments:

We thank the reviewers and the editor for the opportunity to revise our manuscript. Taking the reviewers' comments and recommendations into consideration, we modified the manuscript and changes are highlighted yellow in the revised manuscript. We include with this manuscript, additional data in Figure 3C, a phylogenetic tree in Figure 6, an updated model in Figure 7, and additional views of *Ng* HpaC and consensus sequence analysis in Figure S4. These changes helped us improve the quality of the manuscript.

Point-by-point responses to major issues:

Reviewer #1:

In the discussion (line 317-322), the authors postulate that the FAD reductase HpaC could be co-expressed with the known antibiotic resistance regulator, FarR, in an operon. FarR itself is regulated by the antibiotic resistance regulator MtrR which has also been shown to regulate pili expression (J Bacteriol. 2007 May 4;189(13):4569–4577). I feel that if the authors could show that HpaC and FarR are in an operon, they can improve the sophistication of their model in Figure 5 by incorporating the known regulatory pathway of mtrR controlling both pili expression and HpaC. I feel this would improve the impact of the paper.

Thank you for mentioning this connection between piliation and MtrR. While it is beyond the scope of this manuscript and, therefore, not included in the model, we did include this information in the Discussion in lines 345-347: "MtrR also regulates the expression of the *pilMNOPQ* operon, encoding parts of the T4p secretion system (Folster *et al.* 2007). Therefore, HpaC could be part of the MtrR and FarR transcriptional antimicrobial response that coordinates with piliation status."

Reviewer #2:

I don't have any experiments that need doing. However, it would be interesting to search Gc genome databases to examine naturally occurring polymorphisms in HpaC, and see if any map to similar sites or might be predicted to give a loss of function.

We analyzed the Gc consensus HpaC peptide sequence from ~1000 isolates from PubMLST and noticed that the protein, including glycine-93, is highly conserved. We included this analysis in a new Figure S4E. We also include new complementation data for HpaC(Gly93Ala) in Figure 3C that shows partial complementation.

Reviewer #3:

It is not quite convincing that the hpaC pilE double mutant is not different from the pilE and hpaC single mutants for H2O2 resistance (Fig 4B). The authors should tone down their claims of no effect in the double mutant in lines 193 and 284.

While the double mutant was more sensitive than either single mutant, there was no statistical significance. We have generalized the conclusions in the title of this section by removing T4p dependency to "HpaC promotes LL-37 and H_2O_2 resistance", and we included in lines 200-203 "While the double mutant was more sensitive to H_2O_2 than either single mutant (Figs 4B and S1D), these differences were not statistically significant (p-value >0.99)."

Point-by-point responses to minor issues:

Reviewer #1:

In Figure 1 panel C, I think the last two panels are mislabelled as T1 and T2 and are meant to be T3 and T4.

Thank you for this correction. We've updated Figure 1.

Table 3 and 4: I do prefer to see this information in phylogenetic trees as they are usually more visually memorable than a Table. This is not an essential suggestion but it could look better in the final publication.

Thank you for this suggestion. We include a phylogenetic tree as Figure 6. As a result of this recommendation, we came to an interesting conclusion that *hpaB* or *hpaC* gene loss occurred multiple times during evolution. This finding is now in the Results and Discussion.

FAD reductases have known roles in antibiotic resistance, the most well known of these being the flavin reductases involved in metronidazole sensitivity. To help with the global impact of the observation made here I would have liked to see some mention of this as it would draw readers from other areas and not just Neisseria.

We considered the suggestion to include a section on FAD reductases in antibiotic resistance but we decided that it is not within the scope of this manuscript. While FAD reductases have roles in antibiotic resistance such as with tetracycline and sulfonamide, they involve a monooxygenase. We show that *Neisseria* lacks the monooxygenase HpaB and we do not know if HpaC works with an undefined monooxygenase. The roles of flavoproteins in metronidazole sensitivity are either indirect such as by scavenging oxygen or not applicable to our understanding of HpaC. For instance, metronidazole is a prodrug that requires activation by reduction, while hydrogen peroxide and LL-37 do not, suggesting that HpaC works differently than the flavoproteins in metronidazole activation.

Reviewer #2:

I suggest that the authors are more careful with the title, and also the summary figure. The authors do not demonstrate that HpaC is a flavin reductase. They might think this is the case, and could strengthen this by more careful comparison of its predicted structure with characterized related enzymes. However in the absence of biochemical data, they shouldnt state this in the title based on the evidence presented in the article, and the summary figure should include a ?mark.

Based on the reviewer's comment, we changed the title to "Flavin affinity for the reductase HpaC differentially sensitizes *Neisseria gonorrhoeae* during Type IV pilus-dependent killing"

I think T3 and T4 are incorrectly labeled in Fig 1C as T1 and T2 (again). Also change 'treated with SPRN' to 'backcrossed with SPNR' as it sounds like the gDNA was in the assay. And based on genome sequencing are T3 and T4 identical? If so, say so.

We corrected the labeling in Figure 1C and changed the annotation on the graph to simply "Backcrossed". T3 and T4 are not identical. We reassessed the genome sequencing and identified different phase variations in T3 and T4 and are included in a revised Table 2.

The discussion/analysis of the Alphafold models should be improved. What are the active site residues in this class of enzymes? Are they conserved in Gc? These sies should be shown zoomed in. They should overlay the known and predicted structures to give confidence to the reader of how closely related they are. How often were simulations run? Where is the substitution in relation to FAD? A single view of the structure is not helpful.
We thank this reviewer for their comments. We have included a supplementary figure that highlights the FAD binding loops, several views of Gly-93 and the FAD and NADH binding pocket, and provided an overlay of TftC and HpaC, as the reviewer suggested (Figure S4). Additionally, we included a section in the Methods (lines 559-562): "Aligning FA1090 HpaC to *B. cepacia* TftC. We aligned the peptide sequence of FA1090 HpaC to TftC from *B. cepacia* (PDB 3K88). We used the "align" command in PyMOL with five refinement cycles for outlier rejection. The analysis yielded an alignment with a root mean square deviation (RMSD) 0.684 Å for the backbone Cα atoms."

Authors should be careful re the use of the term binding affinities or binding when affinities/interaction alone would be sufficient as it includes off as well as on rates during the association of the protein with its substrate. For example, the substitution could trap the product (assumed to be $FADH_2$) or indeed limit catalysis so increase association with the substrate. Neither of these mechanisms affect binding per se
We have made the necessary changes in the manuscript to emphasize affinity and interaction over binding.

The authors should discuss why the screen did not identify HpaC loss of function nonsense mutants. These should be much more common than a non-synonymous SNP that alters protein function.
To the best of our knowledge, there is no evidence to support that a non-synonymous mutation is necessarily rarer than a nonsense mutant. As a result, we decided to not include this perspective in the discussion.

Tables 3/4 The parameters of searches should be described in detail (ie protein or nucleotide, cutoffs for identity/similarity and for %coverage, whether they were reiterative, of what databases specifically etc). In this way, it will be clear to the reader whether the absence of HpaB is correct or is due to searches performed with excessive stringency.
We included additional information to the Methods section (lines 556-573) for better clarity: "We searched for the terms "*hpaC*" and "*hpaB*" on the National Center for Biotechnology Institute Gene database [39], accessed each genus listed in the taxonomic groups of Betaproteobacteria (26 genera) and Gammaproteobacteria (57 genera), confirmed the presence of HpaC and HpaB by protein sequence homology, and analyzed the genomic context surrounding either *hpaC* or *hpaB* (~10 genes up or downstream) to identify if the other gene was present. We manually curated the genera into categories of genomes having either *hpaC*, *hpaB*, or both. After ClustalW alignment of the 16S rRNA sequences, we generated neighbor-joining rooted phylogenetic trees in Jalview."

Minor comments:
l61 opacity proteins
Thank you for this correction.

l69 which neutrophil membrane
We've now specified that the "…NADPH oxidase assembles on the neutrophil phagosomal and plasma membranes…" in Line 72.

l134/Fig 1C I dont think it is correct that the 'two transformants... were more resistant to SPN when we repressed pilE than without repression'. The relative surivival of both mutants went down on repression of pilE. This sentence could be split in two.
We agree and split the sentence into two. Lines 138-140 is now "Two transformants (T1 and T2 in Fig 1C) showed similar relative survival as the untransformed parental strain. Interestingly, two transformants (T3 and T4 in Fig 1C) were less sensitive to SPN than transformants T1 and T2."

Reviewer #3:
1. Line 52. Delete the word "main". Gc is the only cause of "gonorrhea".
Thank you. We meant to include the fact that there is a *N. meningitidis* clade that produces symptoms that are indistinguishable from gonorrhea, opening to a possible second cause. Perhaps, this point can be saved for another opportunity.

2. Line 53. Correct to "second leading bacterial sexually transmitted infection…". Viral STIs are more common.
Thank you for this correction.

3. Line 59. While the sentence about macrophage PRRs responding to Gc peptidoglycan and OM components is correct, it leaves the impression that those are the only cells responding. Epithelial cells also respond to those components and call for the neutrophil infiltration.
We now include the role of epithelial cells in neutrophil influx. The sentence (lines 62-64) is the following: "Mucosal epithelial cells and macrophage pattern recognition of shed peptidoglycan fragments and components of the outer membrane trigger neutrophil recruitment to the site of infection…" while referencing Escobar *et al.* 2018 and Kaparakis *et al.* 2010.

4. Line 78. It is the pilus that is assembled and disassembled, whereas this sentence makes it sound like the PilE protein is being taken apart. Reword.
We have revised this section for better clarity. Lines 77-78 in the Introduction now reads
We edited the original sentence to clarify that it is the pilus that assembles and disassembles. We also changed lines 86-87 to "In the absence of the gene *pilE* that encodes the major pilus subunit, pilin, Gc cells are sensitive to antimicrobial peptide LL-37 and H_2O_2."

5. Lines 100-101. It is 1/100 to 1/1000 pilE Gc that survive SPN, not that many that are killed. Similarly, it is 1/10 WT Gc that survive SPN.
Thank you for this correction. The sentence (lines 103-104) is now the following: "This SPN treatment dose and duration results in the relative survival of between 10^{-2} to 10^{-3} Δ*pilE* cells compared to ≥10^{-1} piliated, parental cells."

6. Line 110. It seems odd that adjacent genes lgtC and lgtD would have such different NGO numbers, since the genes were numbered by chromosomal location. Can NGO11620 really be the gene next to NGO2158. Check the numbers.
After reevaluating the phase variation site, we have confirmed that *lgtC* is indeed NGO11620 and *lgtD* is NGO2158. More importantly, we also realized that there was an error with the annotated open reading frame for NGO2158 *lgtD*. The gene starts approximately 340 bp 5' of the previously thought start codon making the phase variation an intragenic variation not intergenic. We have updated the relevant sections in the Results and in Tables 2 and S1.

7. Lines 134-136. This sentence is ambiguous in terms of what two values are being compared (pilE repressed vs unrepressed or resistance between mutants). I know they meant the latter, but the sentence is confusing and imprecise.
Yes, this sentence was unclear. We have re-written this part of the Results (lines 138-140) to the following: "Two transformants (T1 and T2 in Fig 1C) showed similar relative survival as the untransformed parental strain. Interestingly, two transformants (T3 and T4 in Fig 1C) were less sensitive to SPN than transformants T1 and T2."

8. Line 148. Spell "pilE".
Thank you for this correction.

9. Line 178. Since the authors used IPTG to repress genes (through CRISPRi) in the previous figures, it would be helpful to indicate in the legend or the figure (Fig. 3) that IPTG is being used to induce expression of the hpaC complement in this experiment.
Thank you, we modified the figure legend to mention IPTG.

10. The label in Fig. 1C is probably meant to read "T1, T2, T3, T4".
Thank you for this correction.

11. The legend for the model would be more helpful if it was more descriptive of the figure and the conclusions of the study. Also, what are the blue dots supposed to represent?
We include a new figure (Figure 7) that consists of the main findings in the study accompanied by a more descriptive legend.
